# Supplementary material for: Distinct dynamic connectivity profiles promote enhanced conscious perception of auditory stimuli
Source: Commun Biol. 2024 Jul 12;7:856. doi: 10.1038/s42003-024-06533-7 (PMC11245546; doi:10.1038/s42003-024-06533-7)
Supplement: Supplementary file 2 — Supplementary Material [file 42003_2024_6533_MOESM2_ESM.pdf]

# Supplementary Text

## Relationship between pattern occurrence and subjective ratings

We explored the relationship between the brain patterns and participants’ subjective mental states. We asked participants to rate their level of tiredness, success, and attentional focus on a scale of 1-7 after each experimental block. We analyzed the link between participants' ratings and the occurrence probability of each pattern in a given block using Spearman correlations (Figure S2). Our results revealed a significant correlation between feelings of tiredness and the occurrence of Pattern 4 (*rho* = 0.3, $p = 0.004$, after FDR correction. Additionally, there was a weaker correlation between tiredness and the occurrence of Pattern 2 (*rho* = 0.2, $p = 0.048$) and Pattern 5 (*rho* = - 0.21, $p = 0.045$). Moreover, the occurrence probabilities of Pattern 1 (*rho* = 0.21, $p = 0.045$) and Pattern 3 (*rho* = 0.24, $p = 0.026$) were positively correlated with the subjective ratings of success and did not vary with changes in tiredness or attentional focus.

## Supplementary Figures and Tables

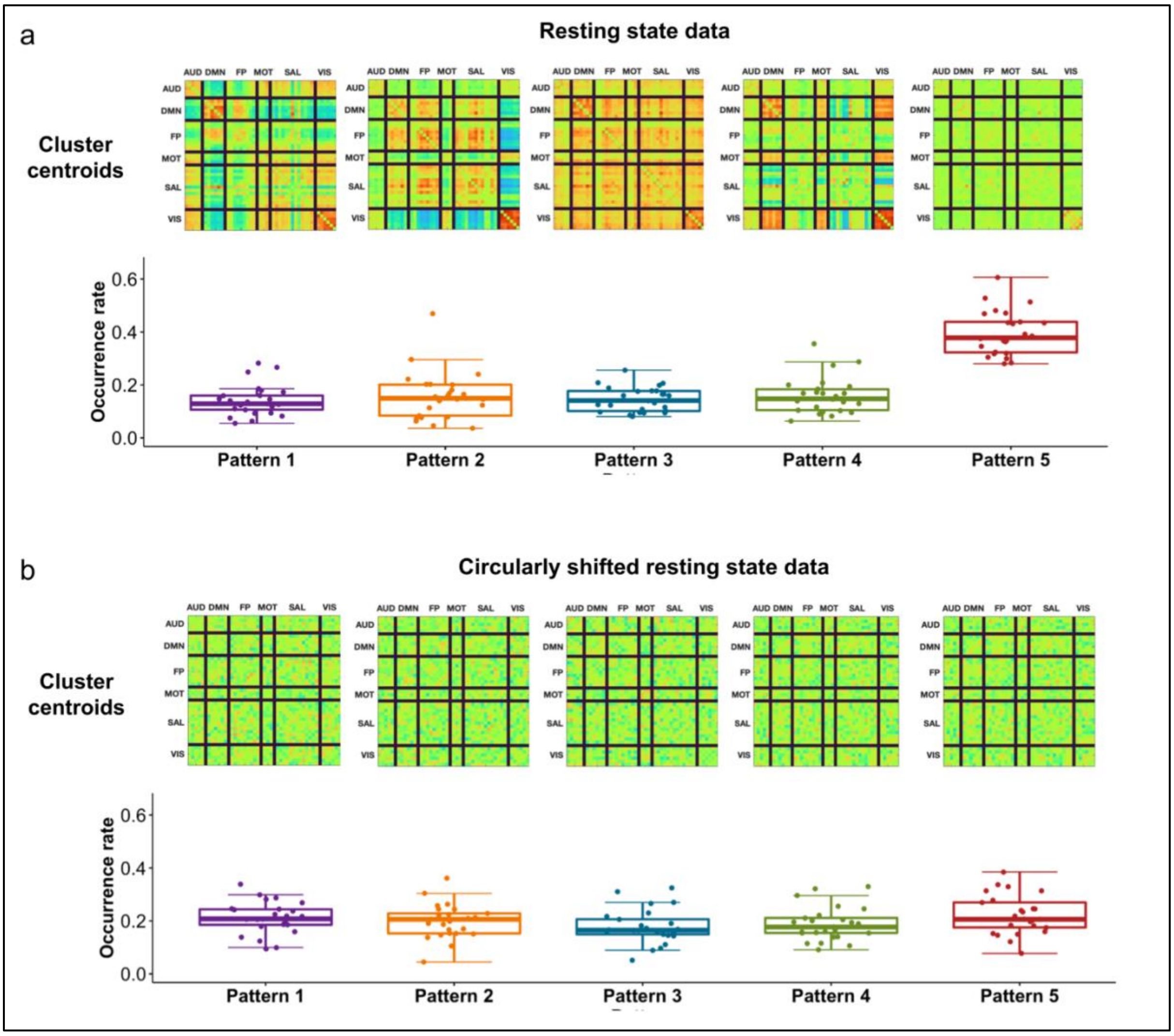


**Supplementary Figure 1. Clusters identified in the original and the surrogate data.** Cluster centroids and their respective occurrence probabilities found using the experimental data (a) and surrogate data (b). The clustering procedure applied to the surrogate data resulted in similar cluster centroids that lacked all types of coherence, providing evidence that the patterns identified in the original data were reflective of the brain's connectivity configurations rather than artifacts introduced by the methodology.

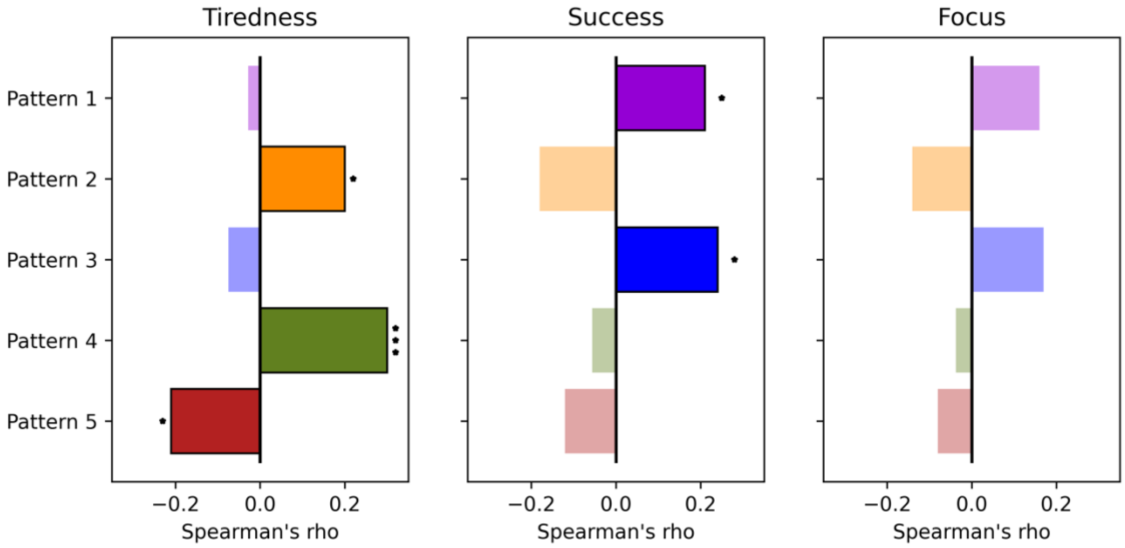


**Supplementary Figure 2. Correlations between post-block subjective ratings and pattern occurrence probabilities.** Spearman correlation values (x-axis) for subjective ratings of tiredness (left panel), success (middle panel), and attentional focus (right panel). Significant correlations after an FDR correction are marked with stars. Non-significant correlation values are shown as translucent.

**Supplementary Table 1. 42 regions of interest used in the inter-areal coherence analyses.** The regions were defined as 10mm-diameter spheres around the given x, y, z coordinates and taken from Demertzi et al. (2019)

| Region of Interest (ROI) | Seed MNI coordinates [x, y, z] |
|---|---|
| **Auditory Network (AUD)** | |
| Anterior cingulate cortex | [6, -7, 43] |
| Precentral gyrus [left] [right] | [-53, -6, 7] [58, -6, 11] |
| Superior transverse temporal gyrus [left] [right] | [-44, -6, 11] [44, -6, 11] |
| **Default Mode Network (DMN)** | |
| Inferior temporal cortex [left] [right] | [-61, -24,-9] [58, -24, -9] |
| Lateral parietal cortex [left] [right] | [-46, -66, 30] [49, -63, 33] |
| Medial prefrontal cortex | [-1, 54, 27] |
| Posterior cingulate cortex | [0, -52, 27] |
| **Fronto Parietal Network (FP)** | |
| Angular gyrus [left] [right] | [-31, -59, 42] [30, -61, 39] |
| Midcingulate cortex | [0, -29, 30] |
| Premotor cortex left [left] [right] | [-41, 3, 36] [41, 3, 36 ] |
| Inferior parietal lobule [left] [right] | [-51, -51, 36] [51, -47, 42] |
| Dorsolateral prefrontal cortex [left] [right] | [-43, 22, 34] [43, 22, 34] |
| **Motor Network (MOT)** | |
| Supplementary motor area | [0, -21, 48] |
| Primary motor cortex [left] [right] | [-39, -26, 51] [38, -26, 48] |
| **Saliency Network (SAL)** | |
| Dorsolateral prefrontal cortex [left] [right] | [-38, 52, 10] [30, 48, 22] |
| Ventrolateral prefrontal cortex | [42, 46, 0] |
| Parietal operculum [left] [right] | [-60, -40, 40] [58, -40, 30] |
| Supplementary motor area [left] [right] | [-5, 14, 48] [5, 14, 48] |
| Dorsal anterior cingulate | [-6, 18, 30] |
| Paracingulate cortex | [0, 44, 28] |

| | |
|---|---|
| Temporal pole [left] [right] | [-50, 14, -14] [51, 16, -19] |
| Orbital frontoinsula [left] [right] | [-40, 18, -12] [42, 10, -12] |
| **Visual Network (VIS)** | |
| Associative visual cortex [left] [right] | [30, -89, 20] [-30, -89, 20] |
| Secondary visual cortex [left] [right] | [-6, -78, -3] [6, -78, -3] |
| Primary visual cortex [left] [right] | [-13, -85. 6] [8, -82, 6] |
