# Supplementary material for: Distinct dynamic connectivity profiles promote enhanced conscious perception of auditory stimuli
Source: Commun Biol. 2024 Jul 12;7:856. doi: 10.1038/s42003-024-06533-7 (PMC11245546; doi:10.1038/s42003-024-06533-7)
Supplement: Supplementary file 3 — reporting summary [file 42003_2024_6533_MOESM3_ESM.pdf]

nature portfolio

Corresponding author(s): Başak Türker

Last updated by author(s): Jun 14, 2024

Reporting Summary

Nature Portfolio wishes to improve the reproducibility of the work that we publish. This form provides structure for consistency and transparency in reporting. For further information on Nature Portfolio policies, see our Editorial Policies and the Editorial Policy Checklist.

Statistics

For all statistical analyses, confirm that the following items are present in the figure legend, table legend, main text, or Methods section.

n/a	Confirmed	
☐	☒	The exact sample size (*n*) for each experimental group/condition, given as a discrete number and unit of measurement
☐	☒	A statement on whether measurements were taken from distinct samples or whether the same sample was measured repeatedly
☐	☒	The statistical test(s) used AND whether they are one- or two-sided<br>*Only common tests should be described solely by name; describe more complex techniques in the Methods section.*
☐	☒	A description of all covariates tested
☐	☒	A description of any assumptions or corrections, such as tests of normality and adjustment for multiple comparisons
☐	☒	A full description of the statistical parameters including central tendency (e.g. means) or other basic estimates (e.g. regression coefficient) AND variation (e.g. standard deviation) or associated estimates of uncertainty (e.g. confidence intervals)
☐	☒	For null hypothesis testing, the test statistic (e.g. *F*, *t*, *r*) with confidence intervals, effect sizes, degrees of freedom and *P* value noted<br>*Give P values as exact values whenever suitable.*
☒	☐	For Bayesian analysis, information on the choice of priors and Markov chain Monte Carlo settings
☒	☐	For hierarchical and complex designs, identification of the appropriate level for tests and full reporting of outcomes
☐	☒	Estimates of effect sizes (e.g. Cohen's *d*, Pearson's *r*), indicating how they were calculated

Our web collection on statistics for biologists contains articles on many of the points above.

Software and code

Policy information about availability of computer code

Data collection	Psychtoolbox-3 and Matlab R2019
Data analysis	- SPM 12, FSL and PhysIO for fMRI preprocessing and denoising<br>- Matlab R2023b and R 2023.09.1+494 for behavioral data analysis and statistics

For manuscripts utilizing custom algorithms or software that are central to the research but not yet described in published literature, software must be made available to editors and reviewers. We strongly encourage code deposition in a community repository (e.g. GitHub). See the Nature Portfolio guidelines for submitting code & software for further information.

Data

Policy information about availability of data

All manuscripts must include a data availability statement. This statement should provide the following information, where applicable:

- Accession codes, unique identifiers, or web links for publicly available datasets
- A description of any restrictions on data availability
- For clinical datasets or third party data, please ensure that the statement adheres to our policy

All data that support the findings of the study as well as the custom analysis scripts can be
573 found in OSF (https://osf.io/fc29k/?view_only=02e409eea0144e069896ea96370afa85).

Research involving human participants, their data, or biological material

Policy information about studies with human participants or human data. See also policy information about sex, gender (identity/presentation), and sexual orientation and race, ethnicity and racism.

Reporting on sex and gender	We recruited 13 women and 12 men for a balanced sample. We did not perform any analysis based on sex or gender
Reporting on race, ethnicity, or other socially relevant groupings	No participant selection was made based on race, ethnicity or any other socially relevant critaria
Population characteristics	Healthy native French speaker participants with good hearing and without any neurological or psychiatric disorders.
Recruitment	Participants were recruited through an online advertisement
Ethics oversight	The protocol had been approved by the local ethics committee (promoted by the INSERM, CPP Ile-de-France 6, C13-41).

Note that full information on the approval of the study protocol must also be provided in the manuscript.

Field-specific reporting

Please select the one below that is the best fit for your research. If you are not sure, read the appropriate sections before making your selection.

☒ Life sciences ☐ Behavioural & social sciences ☐ Ecological, evolutionary & environmental sciences

For a reference copy of the document with all sections, see nature.com/documents/nr-reporting-summary-flat.pdf

Life sciences study design

All studies must disclose on these points even when the disclosure is negative.

Sample size	25 participants were recruited for the study based on a previously published paper
Data exclusions	One participant was excluded from the study due to
Replication	We did not perform any additional replication study on our data
Randomization	The presentation of stimuli was randomized. All participants went through all 4 conditions (3 different SNR levels and catch trials)
Blinding	The experimenter was not aware of the order of stimuli during the presentation

Reporting for specific materials, systems and methods

We require information from authors about some types of materials, experimental systems and methods used in many studies. Here, indicate whether each material, system or method listed is relevant to your study. If you are not sure if a list item applies to your research, read the appropriate section before selecting a response.

Materials & experimental systems

n/a	Involved in the study	
☒	☐	Antibodies
☒	☐	Eukaryotic cell lines
☒	☐	Palaeontology and archaeology
☒	☐	Animals and other organisms
☒	☐	Clinical data
☒	☐	Dual use research of concern
☒	☐	Plants

Methods

n/a	Involved in the study	
☒	☐	ChIP-seq
☒	☐	Flow cytometry
☐	☒	MRI-based neuroimaging

Plants

Seed stocks	*Report on the source of all seed stocks or other plant material used. If applicable, state the seed stock centre and catalogue number. If plant specimens were collected from the field, describe the collection location, date and sampling procedures.*
Novel plant genotypes	*Describe the methods by which all novel plant genotypes were produced. This includes those generated by transgenic approaches, gene editing, chemical/radiation-based mutagenesis and hybridization. For transgenic lines, describe the transformation method, the number of independent lines analyzed and the generation upon which experiments were performed. For gene-edited lines, describe the editor used, the endogenous sequence targeted for editing, the targeting guide RNA sequence (if applicable) and how the editor was applied.*
Authentication	*Describe any authentication procedures for each seed stock used or novel genotype generated. Describe any experiments used to assess the effect of a mutation and, where applicable, how potential secondary effects (e.g. second site T-DNA insertions, mosiacism, off-target gene editing) were examined.*

Magnetic resonance imaging

Experimental design

Design type	Task (event-related design) and resting state
Design specifications	Each participant went through a single fMRI session containing a staircase procedure, an auditory detection task, a resting state recording and an anatomical scan
Behavioral performance measures	Responses to auditory stimuli collected via button-presses. Auditory stimuli detection rates and reaction times are computed and compared between conditions using linear mixed models

Acquisition

Imaging type(s)	Functional and structural
Field strength	3T
Sequence & imaging parameters	All MRI data were acquired on a 3T Siemens Prisma System. T2*-weighted whole brain resting state images were acquired with a multi-band gradient-echo planar imaging (EPI) sequence (600 volumes, 48 slices, slice thickness: 3 mm, TR/TE: 1000 ms/30 ms, voxel size: 3 × 3 × 3 mm, multiband acceleration factor: 3, flip angle: 60°). Functional MRI images during the detection task were acquired using the same sequence. The cardiac and respiratory activities were also recorded during the fMRI acquisitions. An anatomical volume was acquired using a T1-weighted MPRAGE sequence in the same acquisition session (192 or 256 slices, slice thickness: 1 mm, TR/TE: 2.300 ms/ 2.76 ms, voxel size: 1 × 1 × 1 mm, flip angle: 9°).
Area of acquisition	Whole brain scan

Diffusion MRI ☐ Used ☒ Not used

Preprocessing

Preprocessing software	SPM and FSL (topup for distorsion correciton)
Normalization	Normalized though affine transformation
Normalization template	Normalized to the MNI space
Noise and artifact removal	The preprocessing included segmentation using CAT12, realignment, co-registration, and normalization into the MNI152 (Montreal Neurological Institute) space as implemented in SPM12. We did not perform slice-timing correction as our TR was already short (1 second). We also avoided spatial smoothing of our data. The susceptibility-induced off-resonance field distortions were corrected using the topup procedure as implemented in FSL, providing a more accurate representation of the brain. Peripheral physiological data recorded during the scans such as respiration and cardiac pulsation were extracted using PhysIO Toolbox. White matter masks, realignment parameters as well as their first and second order derivatives, cardiac and respiratory signals were included as nuisance regressors in the generalized linear model (GLM) in order to denoise the data.
Volume censoring	No censoring

Statistical modeling & inference

Model type and settings	No activation analyses were made
Effect(s) tested	No activation analyses were made

Specify type of analysis: ☒ Whole brain ☐ ROI-based ☐ Both

Statistic type for inference (See Eklund et al. 2016): No activation analyses were made

Correction: FDR

Models & analysis

n/a	Involved in the study
☐	☒ Functional and/or effective connectivity
☐	☐ Graph analysis
☐	☐ Multivariate modeling or predictive analysis

Functional and/or effective connectivity: Phase-based functional conenctivity using the Hilbert transform of the fMRI timeseries

Graph analysis: -

Multivariate modeling and predictive analysis: -
